# Implementation of community-based adherence clubs for stable antiretroviral therapy patients in Cape Town, South Africa

**DOI:** 10.7448/IAS.18.1.19984

**Published:** 2015-05-27

**Authors:** Anna Grimsrud, Joseph Sharp, Cathy Kalombo, Linda-Gail Bekker, Landon Myer

**Affiliations:** 1 Division of Epidemiology & Biostatistics, School of Public Health & Family Medicine, University of Cape Town, Cape Town, South Africa; 2 The Desmond Tutu HIV Centre, Institute of Infectious Disease and Molecular Medicine, University of Cape Town, Cape Town, South Africa; 3 Gugulethu Community Health Centre, Provincial Government of the Western Cape, Cape Town, South Africa; 4 Department of Medicine, University of Cape Town, Cape Town, South Africa

**Keywords:** models of care, ART delivery, community-based, loss to follow-up, decentralization, task shifting

## Abstract

**Introduction:**

Community-based models of antiretroviral therapy (ART) delivery have been recommended to support ART expansion and retention in resource-limited settings. However, the evidence base for community-based models of care is limited. We describe the implementation of community-based adherence clubs (CACs) at a large, public-sector facility in peri-urban Cape Town, South Africa.

**Methods:**

Starting in May 2012, stable ART patients were down-referred from the primary care community health centre (CHC) to CACs. Eligibility was based on self-reported adherence, >12 months on ART and viral suppression. CACs were facilitated by four community health workers and met every eight weeks for group counselling, a brief symptom screen and distribution of pre-packed ART. The CACs met in community venues for all visits including annual blood collection and clinical consultations. CAC patients could send a patient-nominated treatment supporter (“buddy”) to collect their ART at alternate CAC visits. Patient outcomes [mortality, loss to follow-up and viral rebound (>1000 copies/ml)] during the first 18 months of the programme are described using Kaplan–Meier methods.

**Results and Discussion:**

From June 2012 to December 2013, 74 CACs were established, each with 25–30 patients, providing ART to 2133 patients. CAC patients were predominantly female (71%) and lived within 3 km of the facility (70%). During the analysis period, 9 patients in a CAC died (<0.1%), 53 were up-referred for clinical complications (0.3%) and 573 CAC patients sent a buddy to at least one CAC visit (27%). After 12 months in a CAC, 6% of patients were lost to follow-up and fewer than 2% of patients retained experienced viral rebound.

**Conclusions:**

Over a period of 18 months, a community-based model of care was rapidly implemented decentralizing more than 2000 patients in a high-prevalence, resource-limited setting. The fundamental challenge for this out of facility model was ensuring that patients receiving ART within a CAC were viewed as an extension of the facility and part of the responsibility of CHC staff. Further research is needed to support down-referral sooner after ART initiation and to describe patient experiences of community-based ART delivery.

## Introduction

Models of care for antiretroviral therapy (ART) delivery have evolved considerably over the past decade of ART provision in resource-limited settings. Services have been decentralized from hospitals to primary care facilities and tasks shifted from doctors to nurses and community health workers (CHWs) [1]. However, despite these developments, it is estimated that 95% of HIV services are presently provided within health facilities [2,3]. To support the ambitious targets and accelerated pace of ART expansion and reduce congestion at busy health facilities, further adaptations and health systems changes are needed [3,4]. To this end, decentralizing at least 30% of HIV services into communities has been recommended [3].

Community-based models of care for stable patients present one model of decentralization, designed to make ART delivery more efficient for the health system and provide appropriate support to encourage long-term retention of patients [5]. However, the evidence base for community-based models of care, where treatment, care and support is located outside of health facilities, is limited. In the recent systematic review on decentralization, the three community-based models of care were limited to programmes providing home-based ART delivery [6–10]. While the results suggest that community models can have comparable outcomes to facility-based models, the scalability of home-based ART models is debatable and such models do not encourage patient self-management, a key level of task shifting required for retention in care [11].

The adherence club model of care was designed to support ART maintenance for groups of stable patients in a CHW-facilitated model with peer-support and increased patient self-management [12,13]. The success of an early pilot project in Cape Town [14], led to the model of care being adopted and widely implemented by the City of Cape Town and Western Cape Government Department of Health (DOH) [12,13]. The community health centre (CHC) in Gugulethu adapted the adherence club model to local conditions by shifting the service away from health facilities to be community based. Stable patients were down-referred to community-based adherence clubs (CACs) and managed by CHWs. Here we describe the implementation, early outcomes and lessons learned from the CACs given the limited evidence base for community-based models and the push to further decentralize ART delivery.

## Methods

Adherence clubs were implemented within the Hannan Crusaid Treatment Centre (HCTC) at the Gugulethu CHC in Cape Town, South Africa. The ART programme at the HCTC is a well-characterized, large, public-sector service that has been described in detail previously [15–17]. The HCTC receives support from the Desmond Tutu HIV Foundation and peer counsellor support from the Sizophila programme [16,18]. In Table 1, the standard of ART care at the CHC is compared with the CAC model. Implementation of the CACs is described in detail covering the six core components of the WHO health systems framework [19].

**Table 1:** Comparison of standard of care and CACs for the management of ART patients

	Standard of care (CHC)	Community-based adherence clubs (CACs)
**Setting**	Clinic based	Community based
**Patient profile**	All ART patients	Stable patients
**Key personnel**	Doctors/nurses	CHW
**Frequency of visits**	2 monthly	2 monthly
**Frequency of clinical consultations**	2 monthly (every visit)	12 monthly
**Location of clinical consultations**	CHC	Community based
**Emphasis of patient contacts**	Detecting clinical complications	Treatment adherence, patient wellness
**Units of care**	Individual patient	Groups of 25–30
**Peer-based support**	No emphasis	Strong emphasis
**Patient self-management**	Minimal emphasis	Strong emphasis
**Frequency of laboratory monitoring**	6–12 monthly	12 monthly
**Management of clinical complications**	On-site	Up-referral to CHC
**ART packing and dispensing**	Packed at the CHC pharmacy, dispensed from pharmacy	Pre-packed by central dispensing unit, dispensed at CAC visit
**Treatment buddy**	Patients attend the CHC and collect ART themselves	ART can be collected by a treatment buddy

### CAC eligibility and referral

ART adherence clubs were designed to decongest facilities and support stable ART patients [12,13]. Stable patients were voluntarily down-referred to the CACs. Patients were considered “stable” if they were adherent on the same ART regimen for >12 months, had two consecutive undetectable (<400 copies/mL) viral loads and did not have any other medical conditions requiring more frequent follow-up. Individual patient counselling at the time of referral described the benefits of CAC model, the visit schedule and how to access clinical care outside of CAC visits as necessary.

### CACs venues

The adherence club model was adapted to reduce congestion in the CHC and to provide accessible ART within the community. The first clubs met in a separate building at the CHC. Starting in May 2013, adherence clubs were transitioned from being facility-based to being located at community venues. From June 2013, the clubs were all CACs meeting at a community-based organization (CBO) facility approximately 300 m from the CHC. From April 2014, CACs relocated to a room within the municipal community centre located approximately 450 m from the CHC. Daily transportation by one of the CHC vehicles is provided for the CAC CHWs, the pre-packed ART, the CAC registers and, on a phlebotomy or clinical club day, the CAC nurse.

### CAC operations

Recruitment for CACs began in May 2012 and the first club met in June 2012. Patients who met the CAC eligibility criteria were identified during their clinical consultation and referred to meet with a club CHW. Each club had approximately 25–30 patients who met every two months (eight weeks). At each CAC visit, there was a group counselling session, a brief symptom screening and distribution of ART, all completed in approximately 60 min. The group counselling sessions, facilitated by the CHW, focused on content relevant to stable patients on ART such as safe conception. The CHW weighed all patients and administered a brief screening questionnaire on systemic well-being. Annual visits for phlebotomy and clinical consultations took longer (maximum three hours) due to the time taken for each patient to be seen by the club nurse. At the four-month club visit monitoring blood samples were drawn and this was repeated annually. A clinical consultation with a nurse occurred at the six-month club visit and was repeated annually.

All of the CAC ART was pre-packed off-site at a central pharmacy. Patients received two months of pre-packed ART at their CAC visit. During the October/November visit, four months of pre-packed ART was distributed to CAC members to support migration over the holiday period [20]. Therefore, most CACs met five times per year. The group counselling session in the October/November CAC visit focused on how to ensure adherence to ART when travelling and how to access ART elsewhere should a patient not return to Gugulethu for any reason.

Patients who were late for their CAC visit had a grace period of five working days to collect their ART and remain in a club. CAC patients who were late for their CAC would return to the CAC CHWs at the CHC for either collection of their ART if they were within the grace period or up-referral to the CHC is they were later than five working days. Patients were also up-referred if they were no longer stable. Instability included development of a condition that required more frequent clinical consultations (e.g. tuberculosis), the need to switch regimens or viral rebound.

A patient-nominated treatment supporter or “buddy” could be sent to every alternate CAC visit to collect the CAC patient's ART. The buddy would arrive at the CAC with the patient's clinic card, collect their ART and the visit would be recorded in the CAC register as a buddy collected visit in place of patient's weight and symptom screen. Patients could nominate more than one buddy and the identity of the buddy was not routinely collected.

### CAC monitoring and evaluation

CAC data were recorded in a paper register with one register per CAC. At each CAC visit, the weight of each patient and results of the brief symptom screen were recorded. A summary of the CAC data aggregated at the level of the CAC was completed by the CHW following each CAC visit and monthly group level reports were compiled for the DOH. Standard individual patient files at the CHC of CAC members were only used at the annual clinical visit. When a patient was down-referred to a CAC, the CAC number was placed on the CHC patient file so that clinicians could determine where the patient was receiving ART.

### Human resources and management

CACs were CHW-managed with support from clinical staff at the CHC. The CHWs had varying levels of education and experience as part of the Sizophila peer-counselling programme [16,18]. Each of the four CHWs were responsible for managing between 12 and 18 CACs. The specific responsibilities of a CAC CHW included calling all CAC patients before their first CAC visit, completing club registers, compiling CAC statistics monthly, completing the brief assessment of CAC patients (weighing and symptom screening) on their CAC day, facilitating the group counselling session on CAC days, checking with the pharmacist to see if pre-packed ART was ready for each CAC patient, tracing any CAC patients who did not arrive and recording blood results in the CAC registers.

A professional nurse was assigned as the CAC nurse rotating on a monthly basis. The CAC nurse was at the community venue for blood and clinical CAC visits approximately two days per week. If a CAC patient needed to see a clinician on their CAC visit date, the patient was referred to the CHC and had priority access to the CAC nurse.

One of the pharmacists at the CHC was responsible for the CACs. The CAC Pharmacist ensured that there was a valid script for each CAC patient and that the scripts included the CAC number and first visit date for dispensing. The central pharmacy delivered the pre-packed medication parcels to the CHC pharmacy three days before the CAC visit date. On the day of the CAC, the pharmacist provided all the pre-packed ART for the CAC to the CHW for distribution to CAC patients.

Management of the CACs was integrated within the CHC management. The CHC clinical manager provided oversight with support from the CHC facility manager. No additional financing was required during the implementation of CACs. In December 2013, a clubs manager was appointed from the existing CAC CHW team and a new CHW was recruited to join the CAC. This position was created to support the growing programme and alleviate the administrative CAC responsibilities previously completed by the clinical manager of the CHC.

### Analysis

Ethical approval for the collection of routine clinic data and data on adherence clubs was obtained from the Human Research Ethics Committee (HREC) in the Faculty of Health Sciences at the University of Cape Town (HREC Refs 359/2002 and 383/2012). Written informed consent was provided by all enrolled patients for anonymous clinical data to be recorded and analyzed. Data were analyzed using STATA 13.0 (STATA Corporation, College Station, Texas, USA). All patients down-referred to an adherence club between May 2012 and December 2013 were included in the analysis. Patients entered the analysis on the date of their first club visit. Patient outcomes were assessed at the end of 2013 (analysis closure). The primary outcome of interest was loss to follow-up (LTFU) with secondary outcomes of mortality and viral rebound also reported. LTFU was defined as having no contact at a CAC or the CHC in the first 12 weeks of 2014. For patients defined as LTFU, the date of last contact was the LTFU date. Viral rebound was defined as having a single viral load measure of >1000 copies/ml after suppression. Patients were censored at the date of death, transfer or LTFU.

The number of new CAC patients and CACs were reported by month. Median year of club initiation is reported biannually and described by pre-ART characteristics. Patient characteristics at ART initiation (sex, age, CD4 cell count, viral load and year of initiation), CAC initiation (age, CD4 cell count, time on ART) and demographics (distance from clinic, employment status) at CAC initiation are described and summarized using appropriate summary statistics. Median time to CAC initiation was calculated by pre-ART characteristics with interquartile ranges (IQRs) and Wilcoxon rank-sum tests used to investigate associations. Time to event analysis was conducted using Kaplan–Meier methods. Kaplan–Meier graphs of the outcomes (mortality, LTFU, viral rebound) are presented as proportions and reported every three months.

## Results

**Figure 1:**
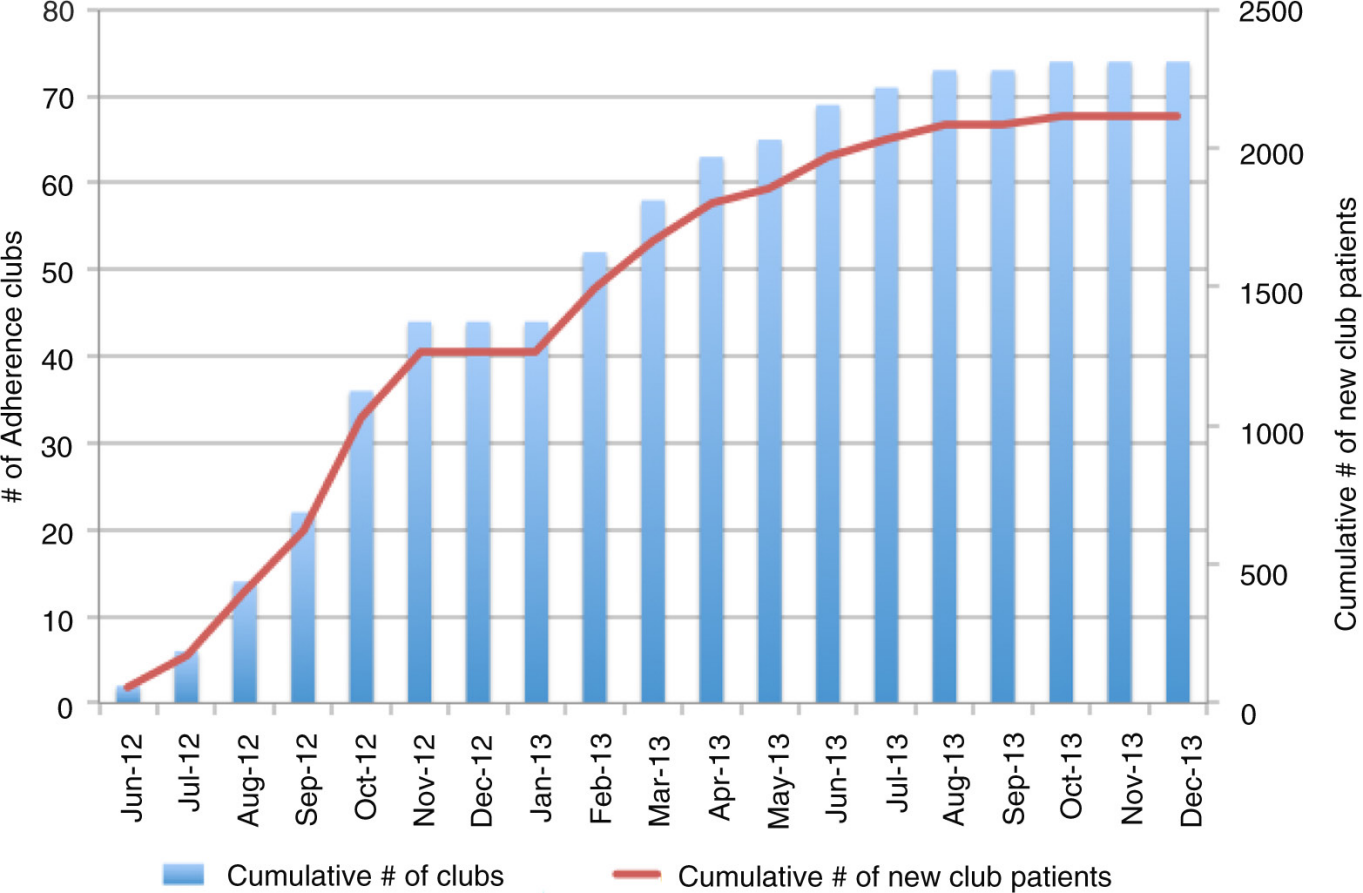
Implementation of community-based adherence clubs between June 2012 and December 2013.

Over an 18-month period, 2113 patients were decentralized to one of 74 CACs (Figure 1). During this same period, 2776 patients were initiated onto ART at the CHC. By December 2013, approximately one-third of the CHC patients were managed in a CAC. CAC patients were predominantly female (71%), lived within 3 km of the CHC (72%) and unemployed (58%) (Table 2). The median pre-ART CD4 cell count was 134 cells/µl and 32% of CAC patients had been on ART for six years or longer at the time of joining a CAC. At the time of joining a CAC, the median age was 39 years and the median CD4 cell count was 517 cells/µl.

**Table 2:** Characteristics and demographics of community-based adherence club patients pre-ART and at time of club start

	Adults (≥16 years)
	*n* = 2113
Gender, *n* (%)	2113 (100)
Female, *n* (%)	1489 (70.5)
Age at club start (years), median (IQR)	38.8 (34.0–44.5)
Age categories at club start (years), *n* (%)	
16–24	38 (1.8)
25–34	593 (28.1)
35–44	974 (46.1)
≥45	508 (24.0)
CD4 cell count at club start (cells/µl), *n* (%)	2109 (99.8)
<200	49 (2.3)
200–399	502 (23.8)
400–599	846 (40.1)
600–799	439 (20.8)
≥800	272 (12.9)
Median (IQR)	517 (396–669)
Pre-ART Viral load, log_10_ copies/ml, *n* (%)	1588 (75.2)
Median (IQR)	4.8 (4.3–5.2)
Years on ART at club start, median (IQR)	4.6 (2.5–6.6)
<1.5 years	211 (10.0)
1.5–3 years	465 (22.0)
3–4.5 years	407 (19.3)
4.5–6 years	347 (16.4)
6–7.5 years	407 (19.3)
≥7.5 years	276 (13.1)
Distance from the CHC, n(%)	1392 (65.9)
<1 km	463 (33.2)
1–3 km	540 (38.9)
3–5 km	254 (19.0)
>5 km	12 (9.1)

The median time from ART initiation to CAC uptake was 4.4 years (IQR 2.5–6.6) (Table 3). For CAC patients who initiated ART in 2011 and 2012 when ACs were available, time to CAC initiation was 1.6 (IQR 1.4–1.9) and 1.2 (IQR 1.1–1.3) years, respectively. The median year of ART initiation among CAC patients increased from 2007 (IQR: 2005–2009) among those joining a CAC during 2012, to 2009 (IQR: 2006–2011) in the first half of 2013 and 2011 (IQR: 2010–2012) and in the latter half of 2013. The median time since first suppressed viral load also changed over time. Patients joining a CAC during 2012 had their first viral load 4.5 years before (IQR: 2.5–6.4), compared to 3.3 years among those joining a CAC in the first half of 2013 (IQR: 1.0–6.0) and 2.1 years among those joining a CAC in the latter half of 2013 (1.2–3.0) (results not shown).

**Table 3:** Median time to community-based adherence club initiation by pre-ART characteristics

Pre-ART characteristic	Median time, years (IQR) (*n*=2113)	*p*
Overall	4.4 (2.5–6.6)	
Gender		
Females	4.5 (2.5–6.7)	0.262
Males	4.3 (2.4–6.5)	
Age (years)		
16–24	4.1 (2.5–6.4)	0.013
25–34	4.8 (2.6–6.7)	
35–44	4.2 (2.5–6.6)	
≥45	3.8 (2.3–6.2)	
CD4 cell count (cells/µl)		
<50	5.9 (3.5–7.2)	<0.001
50–99	5.4 (2.3–7.1)	
100–199	4.8 (2.9–6.5)	
≥200	2.7 (1.7–4.8)	
Missing	3.7 (1.8–6.6)	
Year of initiation		
2002–2004	8.6 (8.2–9.2)	<0.001
2005–2007	6.4 (5.7–7.1)	
2008–2010	3.3 (2.6–4.0)	
2011–2012	1.4 (1.2–1.7)	

**Figure 2:**
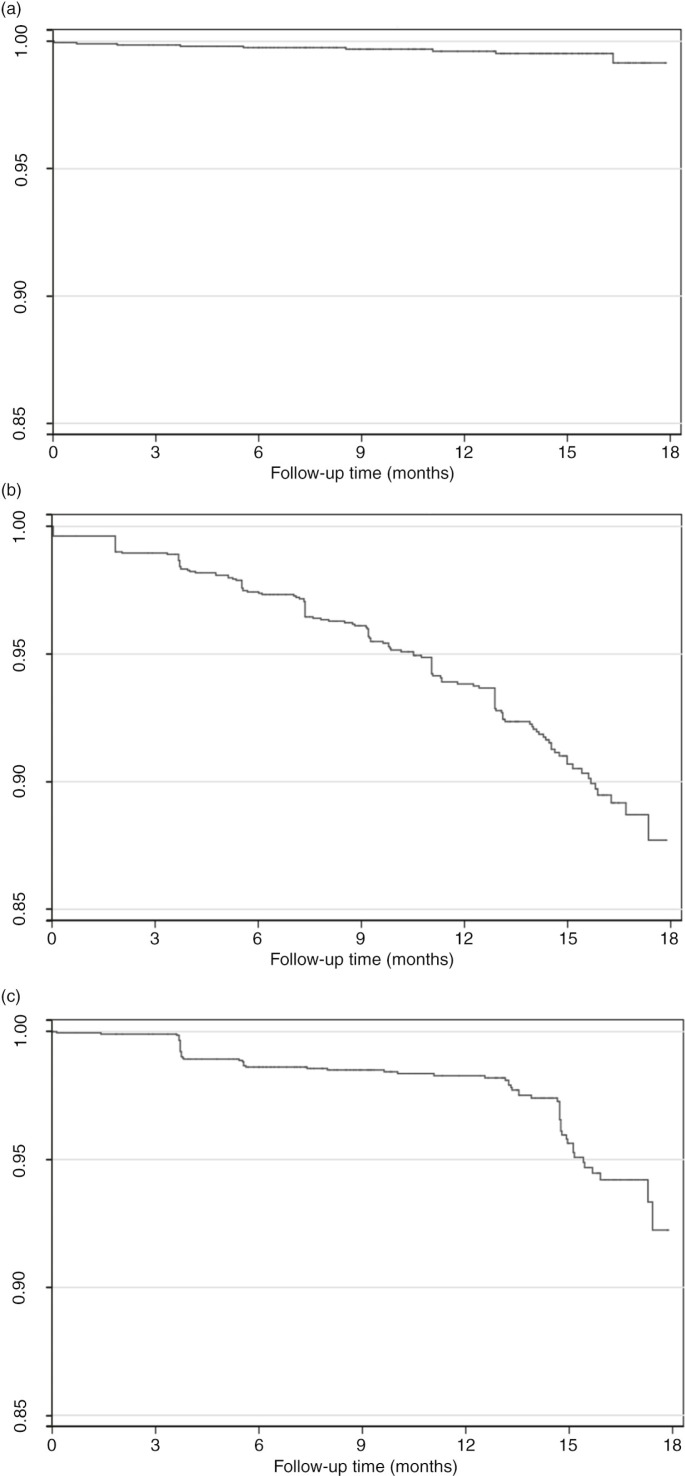
Kaplan–Meier plots of community-based adherence clubs: (a) mortality, (b) loss to follow-up and (c) viral rebound.

During the study period, nine patients in a CAC died (<0.1%) (Figure 2a), 53 were up-referred to the CHC for clinical conditions (0.3%) and 573 CAC patients sent a buddy to at least one club visit (27.1%). Of those 16–24 years of age at club initiation, 13% sent a buddy to at least one club visit compared to 28% among patients 25–34 years of age, 30% among patients 35–44 years of age and 21% among patients 45 years or older at club initiation (*p*-value <0.001). LTFU among CAC patients was 2.6% (95% CI 2.0–3.4), 3.9% (95% CI 3.1–4.8) and 6.2% (95% CI 5.1–7.4) at months 6, 9 and 12, respectively (Figure 2b, Table 4). Kaplan–Meier estimates of viral rebound were 1.4% (95% CI 1.0–2.0) at six months and 1.7% (95% CI 1.2–2.4) at 12 months (Figure 2c, Table 4). Overall retention on ART was 97.2% (95% CI 95.4–97.8) at six months and 93.5% at 12 months (95% CI 92.2–94.5).

**Table 4:** Kaplan–Meier estimates of mortality, loss to follow-up and viral rebound by duration of follow-up after community-based adherence club initiation^a^

Duration of follow-up	*n* (%)	Mortality % (95% CI)	Loss to follow-up % (95% CI)	Viral rebound^b^ % (95% CI)
3 months	2078 (98.3)	0.1 (0.1–0.4)	1.0 (0.7–1.6)	0.1 (0.1–0.4)
6 months	1925 (91.1)	0.2 (0.1–0.6)	2.6 (2.0–3.4)	1.4 (1.0–2.0)
9 months	1602 (75.8)	0.3 (0.1–0.7)	3.9 (3.1–4.8)	1.5 (1.1–2.1)
12 months	1170 (55.4)	0.4 (0.2–0.8)	6.2 (5.1–7.4)	1.7 (1.2–2.4)
15 months	572 (27.1)	0.5 (0.2–1.0)	9.3 (9.9–11.0)	4.4 (3.3–5.8)
18 months	63 (3.0)	0.9 (0.3–2.2)	12.3 (9.7–15.5)	7.8 (5.2–11.6)

a Estimates are from time of community-based adherence club initiation

b Viral rebound is defined as a single viral load measure above 1000 copies/ml after suppression.

## Discussion

Over a period of 18 months, more than 2000 stable ART patients were successfully decentralized from a doctor-driven primary health care clinic to a community-based model of care where they were managed by four CHWs. We adapted the adherence club model to be community-based with all visits occurring out of the facility. CAC patients accessed ART and received annual clinical consults in the community, thus decongesting the primary health care facility. The size and scale up of the CAC model is unprecedented, with implementation occurring more rapidly and the volume of patients much larger than previous models of care described [14,21].

This model of care exemplifies the substantial paradigm shift in ART delivery over the past decade from doctor-led facility-based care towards decentralization of care and task shifting of patient care responsibilities. When ART became publically available in 2004, programmes were largely individualized, hospital based and doctor led [22]. In South Africa, task shifting has included increasing the number of nurses trained to initiate ART from 250 in February 2010 to 23,000 by May 2013 [23]. Concurrently, ART services have been increasingly decentralized with more than 3500 facilities supporting ART provision by mid-2013 [23,24]. The findings of our study highlight that further decentralized community-based models with task shifting to CHWs can successfully support ART maintenance and encourage patient self-management. Increasing patient self-management is crucial for ART programmes to expand and for HIV to be successfully managed as a chronic condition [25].

Patients in the CACs had high levels of retention and virologic suppression. At 12 months, retention on ART was 94%, LTFU was 6% and 2% of patients had experienced viral rebound. While retention in care was less than the 97% reported from the pilot study of facility-based adherence clubs [14], these are promising outcomes. Limited outcome data are available from other community-based programmes with most community models providing home-based ART delivery [26,27]. The exception is the community adherence group (CAG) model in Mozambique with 98% retention in care after 12 months [21]. Retention at 12 months in traditional facility-based models of care is estimated to be 80% [28], but this group is not comparable given that CACs were restricted to stable patients.

Our findings should be considered in light of a number of limitations. It is difficult to determine the generalizability of our findings as the context in which these innovative models of care are implemented is of critical importance. We implemented a novel model of care in a high-prevalence site with an existing CHW programme with considerable NGO support. In addition, the site had a well-functioning pharmacy and logistics system without ART stock-outs during the study period. It is difficult to gauge the transferability of our model to other settings, but our results highlight that its feasibility should be considered elsewhere. Furthermore, flexibilities within the model allow for adaptations to local contexts. Models of ART delivery are not a one-size-fits-all solution [5]. Adaptations to local context will therefore be necessary for broader implementation. The focus of this analysis was limited to early outcomes and more research is necessary to assess factors associated with retention in this model of care and long-term outcomes of decentralized patients. In addition, the impact of decentralizing patients on the workload of the CHC is not assessed.

We determined key factors and challenges that contributed to implementation success (Table 5). A cohesive team comprising the CHWs, pharmacist and clinic management worked as a collective despite having different line managers. It was essential that all clinic staff understood the benefits of the model and trusted that patients could be successfully managed outside of the traditional model of care and clinic facility. Second, policies allowing ART to be distributed within the community were agreed upon by the provincial, sub-district and facility teams and could therefore be used. Policies that impeded implementation were allowing only two months of ART to be dispensed per visit and mandating rescripting of ART every six months. Furthermore, CACs received a reliable supply of ART. Without a reliable drug supply, patients may not trust a community model of care [13]. The CHWs involved in CACs were part of a well-established cadre of staff at the CHC and played a pivotal role in providing quality treatment support to CAC members.

**Table 5:** Key factors and challenges to implementation success of the CAC model

	Factor for implementation success	Challenge for implementation success
**Community-based models as an extension of the facility**	Strong bi-directional referral pathways between facility and community-based models	Patients in community-based models not viewed as the responsibility of the facility (i.e. reluctance to assist with rescripting of CAC patients)
**Location**	Stable patients managed outside of health care facility	Ensuring access to a clean and appropriate community-based facility
**Staffing**	Cohesive, multidisciplinary team including recognized cadre of CHWs	Different categories of staff have different line managers
**ART distribution**	ART distribution by CHWs supported by the pharmacy	Policies regarding dispensing and distribution (i.e. only two months of ART allowed to be dispersed per visit)
**ART supply**	Reliable, uninterrupted supply	Frequent shortages in many areas of the country
**Resources for community-based ART delivery**	CHWs using their personal cell phones	Limited resources within the community venue and distance to CHC for supplies

A fundamental factor to the success of the CACs was that patients receiving ART within the community were regarded as an extension of the CHC with the facility continuing to assume full responsibility and accountability for CAC patients’ care [5]. Since the CHC did not routinely see the patients receiving ART in the community-based model, these patients could easily be forgotten or dismissed as not the responsibility of the CHC. It was difficult to engage staff that were not dedicated CAC staff to support CAC patients in CAC activities such as rescripting. This echoes challenges previously described in the literature where health personnel are trained within a hierarchical framework of health care delivery and do not provide a supportive environment for CHWs [29,30]. Ongoing and sustained efforts are needed to emphasize that CAC patients continue to be CHC patients.

Our experience of implementing community-based ART delivery was not without challenges (Table 5). The location of the CACs within the community had to shift from a CBO facility to a municipal community centre. A long-term standing agreement with the municipal venue is still underway requiring negotiation at the city and the community levels. While in principle the DOH is supportive of expanding community-based ART delivery, in practice there have been significant barriers to expansion. A unique challenge of community-based delivery models comes from the physical location outside of existing health facilities. Any upgrades or maintenance at community venues is not covered within DOH budgeting and it is unclear if extending responsibilities of staff to outside of the CHC is within their contractual agreements. Being located outside of the primary care facility also provides daily logistical challenges due to transportation of materials between the community facility and the primary care facility.

Adaptation of the CAC model will be necessary for further implementation. The criteria for inclusion in CACs requires critical appraisal to determine the optimal selection conditions for CAC inclusion. The frequency of CAC visits, the window period for late ART collection by CAC patients and the type of symptom screening completed at CAC visits can all be adapted to align with local policy. While we utilized a central pharmacy for the CAC ART, this component of the model could be adapted with ART packing completed at the facility level. It is recommended that the CAC model be phased in over time, allowing for both patients and staff to see the benefits while being reassured that the model can adequately support stable patients without undue risk. It is crucial that the CACs and all community-based delivery models be viewed not just as a response to the high patient volume but as beneficial and even preferable to patients [5]. Community-based models of care allow patients to return to normal life and increase their self-management, thus increasing the effectiveness of ART delivery [22,31].

## Conclusions

While there is an urgent need for ongoing research to optimize community-based ART delivery, our implementation findings support continued expansion of community-based models of ART delivery in high-prevalence, resource-limited settings. The adherence club model was successfully adapted, decentralized and implemented to provide community-based care, and our novel model supported ART maintenance exclusively within the community. Additional data and shared experiences from innovative community-based models of care are needed to support long-term ART retention as ART cohorts in resource-limited settings continue to expand and mature.
